# Draft Genome Sequence of Multidrug-Resistant *Mycobacterium tuberculosis* Strain CWCFVRF MDRTB 670, Isolated from the Sputum of a Patient from Chennai, India, with Clinically Suspected Tuberculosis

**DOI:** 10.1128/genomeA.00475-14

**Published:** 2014-05-22

**Authors:** Dhanurekha Lakshmipathy, Umashankar Vetrivel, Lily Therese Irudayam, Gayathri Ramasubban, H. N. Madhavan, R. Sridhar, N. Meenakshi

**Affiliations:** a L&T Microbiology Research Centre, Vision Research Foundation, Chennai, India; b Center for Bioinformatics, Vision Research Foundation, Chennai, India; c Department of Thoracic Medicine, Stanley Medical College, Chennai, India; d Institute of Thoracic Medicine, Chetpet, Chennai, India

## Abstract

We announce the draft genome sequence of a multidrug-resistant *Mycobacterium tuberculosis* strain (CWCFVRF MDRTB 670) isolated from sputum from a patient with clinically suspected tuberculosis.

## GENOME ANNOUNCEMENT

*Mycobacterium tuberculosis* is a highly transmissible bacterial pathogen causing tuberculosis (TB) with an estimated rise of 8.6 million new cases worldwide, of which 3.6% were attributed to multidrug-resistant (MDR) strains (1). Rapid diagnostic methods with accurate susceptibility testing of first-line anti-tuberculous drugs are critical for the early diagnosis and for effective treatment of MDR-TB. Genotypic drug susceptibility testing by PCR-based DNA sequencing analysis studies on *M. tuberculosis* has revealed significant mutations in the drug target genes leading to resistance. Similar studies have also revealed the alteration of drug titer through overexpression of genes coding for drug resistance (2). MDR-TB strains primarily originate due to the accumulation of mutations in multiple drug target genes (3). These mutations, in turn, have become the basis for new rapid, reliable diagnostic tests and have also led to the exploration of new drug targets for the development of novel anti-TB therapeutics. Recent advances in whole-genome sequencing of drug-resistant *M. tuberculosis* strains will enable the development of newer genotypic bases for rapid and highly accurate diagnosis, particularly in developing countries. This will also aid in the exploration of the mechanism of drug resistance in MDR-TB strains.

We announce the draft genome sequence of a multidrug-resistant sputum isolate of *M. tuberculosis*, strain CWCFVRF MDRTB 670, isolated from a patient with clinically suspected tuberculosis. This isolate was resistant to all the first-line anti-tuberculous drugs, streptomycin, isoniazid, rifampin, ethambutol, and pyrazinamide, which was confirmed through phenotypic drug susceptibility testing using a micro BACTEC MGIT culture system. DNA was extracted from the isolate using the sodium dodecyl sulfate extraction method (4) and was further purified using a DNeasy miniprep kit (Qiagen, Hilden, Germany). Whole-genome sequencing was performed using an Ion Torrent (PGM) sequencer. Standard protocols as per the manufacturer’s instructions were followed for library preparation and emulsion PCR (emPCR). The constructed library was quantified using a high sensitivity DNA assay (Agilent Technologies), and recommended concentrations of the library template were used for emPCR. Further, the emPCR product was enriched and loaded onto 316 chips and sequenced using an Ion PGM 400 sequencing kit. Subsequently, the generated sequence reads were filtered with a Phred score cutoff of ≥20. The filtered sequences were *de novo* assembled using MIRA Assembler 3.4.1.1, wherein 261 contigs totalling 4,342,105 bp in length, with 113.38× coverage and an *N*_50_ length of 32,479 bp, were obtained. These sequences were further ordered and reoriented with *M. tuberculosis* H37Rv (accession no. NC_000962.3) as a reference using Mauve (5) and in-house written scripts. Furthermore, the assembled sequences were also subjected to annotation by NCBI PGAAP (http://www.ncbi.nlm.nih.gov/genomes/static/Pipeline.html), which revealed 4,028 protein-coding genes and 56 RNA-coding genes.

The availability of this genome sequence will aid in the better understanding of mutations leading to drug resistance and provide insight into the molecular epidemiology of MDR-TB strains from the local population in Chennai, India.

### Nucleotide sequence accession numbers.

This whole-genome shotgun project has been deposited at DDBJ/EMBL/GenBank under the accession no. JDVY00000000. The version described in this paper is version JDVY00000000.1.
